# The predictive validity of the Strengths and Difficulties Questionnaire in preschool age to identify mental disorders in preadolescence

**DOI:** 10.1371/journal.pone.0217707

**Published:** 2019-06-03

**Authors:** Louise G. Nielsen, Martin K. Rimvall, Lars Clemmensen, Anja Munkholm, Hanne Elberling, Else Marie Olsen, Charlotte Ulrikka Rask, Anne Mette Skovgaard, Pia Jeppesen

**Affiliations:** 1 Child and Adolescent Mental Health Centre, Mental Health Services Capital Region of Denmark, Gentofte, Denmark; 2 Department of Clinical Medicine, Faculty of Health and Medical Sciences, University of Copenhagen, Copenhagen, Denmark; 3 Center for Telepsychiatry, Mental Health Services, Region of Southern Denmark, Odense, Denmark; 4 Department of Clinical Research, University of Southern Denmark, Odense, Denmark; 5 The Department of Paediatrics and Adolescent Medicine, The Juliane Marie Centre, Rigshospitalet, Copenhagen University Hospital, Copenhagen, Denmark; 6 Center for Clinical Research and Disease Prevention, Frederiksberg Hospital, Capital Region of Denmark, Copenhagen, Denmark; 7 Department of Public Health, Faculty of Health and Medical Sciences, University of Copenhagen, Copenhagen, Denmark; 8 Department of Child and Adolescent Psychiatry, Psychiatry Aarhus University Hospital, Aarhus, Denmark; 9 Department of Clinical Medicine, Aarhus University, Aarhus, Denmark; 10 National Institute of Public Health, Faculty of Health Sciences, University of Southern Denmark, Odense, Denmark; Chiba Daigaku, JAPAN

## Abstract

The Strengths and Difficulties Questionnaire (SDQ) is a brief, widely used instrument to screen for mental health problems in children and adolescents. The SDQ predictive algorithms developed for the SDQ, synthesize information from multiple informants regarding psychiatric symptoms and their impact on daily life. This study aimed to explore the validity of the SDQ predictive algorithms used in preschool age to predict mental disorders in preadolescence. The study population comprises 1176 children from the Copenhagen Child Cohort 2000 (CCC2000) assessed at age 5–7 years by the SDQ and reassessed at 11–12 years with the Development and Well Being Assessment (DAWBA) for evaluation of ICD-10 mental disorders. Odds Ratios (ORs), sensitivities, specificities, positive predictive values (PPVs) and negative predictive values (NPVs) were calculated for the SDQ predictive algorithms regarding ICD-10 diagnoses of hyperkinetic-inattentive-, behavioural- and emotional disorders. Significant ORs ranging from 2.3–36.5 were found for the SDQ predictive algorithms in relation to the corresponding diagnoses. The highest ORs were found for hyperkinetic and inattentive disorders, and the lowest for emotional disorders. Sensitivities ranging from 4.5–47.4, specificities ranging from 83.0–99.5, PPVs ranging from 5.0–45.5 and NPVs ranging from 90.6–99.0 were found for the SDQ predictive algorithms in relation to the diagnoses. The results support that the SDQ predictive algorithms are useful for screening at preschool-age to identify children at an increased risk of mental disorders in preadolescence. However, early screening with the SDQ predictive algorithms cannot stand alone, and repeated assessments of children are needed to identify, especially internalizing, mental health problems.

## Introduction

In a recent meta-analysis, the worldwide prevalence of any mental disorder was estimated to 13.4% (95% CI 11.3–15.9) among children and adolescents [1]. Previous longitudinal studies have shown that having a mental disorder at an early age increases the risk of having a mental disorder later in life [2] and mental disorders in early adulthood are often preceded by difficulties in adolescence [3]. However, there is a gap between the number of children who meet criteria for mental disorders in general population studies and the number of children in treatment for mental disorders [4]. The delay from onset of impacting problems to a formal diagnosis and initiation of treatment, i.e. the duration of untreated illness can be vast, and a recent study suggests that this gap might be especially large for mental disorders with early onset [5].

In order to implement timely preventive interventions, screening instruments are needed to identify individuals at an increased risk of developing mental disorders. The Strengths and Difficulties Questionnaire (SDQ) is one of the most frequently used questionnaires to screen for mental health difficulties in childhood and adolescence [6] and has been translated into more than 80 languages. It is a brief questionnaire including 25 questions on mental health strengths and difficulties [7]. These 25 questions are divided into 5 subscales: hyperactivity/ inattention, conduct problems, emotional symptoms, peer relationship problems, and pro-social behaviour [8]. An extended version with questions on daily impact of problems adds important information about the child’s need for intervention [7]. The SDQ can be completed by the children themselves (from age 11), the parents, and/or the teacher [8]. Previous research has found that information from multiple informants is essential in screening for mental health problems in children [9, 10]. For the SDQ specifically a greater sensitivity has been found when using information from both the parent and the teacher compared to using information from just one informant [11].

The SDQ was translated into Danish in year 2001 [12] and has since then been completed by more than 70,000 Danish children in general population-based cohorts [13]. The distributions of the SDQ scores determined in the Nordic countries have been found to be comparable across the Nordic countries [12]. In a review including 48 studies of primary-school-aged-children screened with the SDQ, satisfactory reliability has been reported with acceptable internal consistency, satisfactory test-retest reliability and high inter-rater agreement between teachers and parents [6]. Good validity of the five-factor structure of SDQ was also found, meaning that the SDQ subscales distinguish between different dimensions of psychopathology [6]. There is also ample documentation that the SDQ is a satisfactory screening instrument for concurrent mental disorders [6].

In addition to the well-validated SDQ questionnaire, SDQ predictive algorithms have been developed. The algorithms combine symptoms- and impact scores from several informants, and thereby estimate the risk for being above the diagnostic threshold for a disorder of hyper-activity/inattention, conduct disorders and emotional disorder [11]. The algorithms estimate ‘unlikely’, ‘possible’ and ‘probable’ diagnosis. The SDQ predictive algorithms were originally validated in cross-sectional studies with regard to concurrent diagnoses in a community sample of 8000 5-15-year-olds. Information from multiple informants was included and sensitivities of 86.1% for hyperkinetic disorder, 76.2% for conduct-oppositional disorder, 74.6% for depressive disorder and 50.5% for anxiety disorder were found [11]. The algorithms for hyperactivity-inattention disorder, conduct disorder and emotional disorder have also been validated with regard to concurrent diagnoses in clinical samples in Britain and Bangladesh where higher values were found. Sensitivities of 89% were found for hyperactivity-inattention disorder, sensitivities of 81% in London and 86% in Dhaka were found for emotional disorder and lastly 90% in London and 86% in Dhaka were found for conduct disorder [14]. The SDQ predictive algorithms have afterwards been utilized in several cross-sectional studies [15]. Among these a Norwegian cross-sectional study examined the agreement between the SDQ predictive algorithms and the ICD-10/ DSM-IV diagnoses based on a standardized diagnostic interview on child development and psychopathology (DAWBA; the Development and Well-Being Assessment [16]) for 286 children and adolescents aged 5–18, who were referred to diagnostic assessment. Sensitivities of 0.77 for hyperactivity disorders, 0.83 for conduct disorders, 0.47 for emotional disorders and 0.85 for any disorders were found, while higher values were found for possible cases [17]. Similar and somewhat higher values were found in a high-risk cross-sectional study examining the agreement between the SDQ predictive algorithms and DAWBA diagnoses [18]. Additionally similar values were found in an Australian cross-sectional study examining the agreement between the SDQ predictive algorithms and clinical diagnoses, but with higher values found for conduct disorders and lower values found for hyperactivity disorders [19].

The prognostic value of SDQ has been examined in longitudinal studies, especially in the recent years [20–26]. However, only one longitudinal study included the SDQ predictive algorithms to our knowledge [26]. This Danish study examined register-based ADHD diagnoses in children from the Copenhagen Child Cohort 2000 (CCC2000) and showed that the diagnostic algorithm for ADHD used at age 5 to 7 years could identify a group of children with a 20-fold increased risk of being diagnosed with ADHD in the hospital settings and/or receiving central stimulant medication before age 12 years [26].

The current study is an extension of this former Danish ADHD study and will contribute to the field by broadening the scope and examine the SDQ predictive algorithms’ ability to predict a broad range of mental disorders in children from the general population over a long follow-up period of 6 years using information from both parents and teachers. We investigate children in the key developmental periods: preschool age (5–7 years) and to middle school/ preadolescence (11–12 years) and we explore the predictive properties of the SDQ predictive algorithms as a measure to identify children in preschool who may later fulfil the criteria for one (or several) mental disorder in preadolescence.

## Methods

### Ethical approval

All procedures performed in studies involving human participants were in accordance with the ethical standards of the institutional and/ or national research committee and with the 1964 Helsinki declaration and its later amendments or comparable ethical standards. The CCC2000 study was approved by the Danish Data Protection Agency (RHP-2013-001 I-Suite 02015) and by the Capital Region of Denmark (J. nr 2007-58-0015). The National Committee on Health Research Ethics was consulted (J. nr. H-C-FSP-2010 and KA-05103) in accordance with national guidelines. Participation was voluntary, and data was kept confidential. The parents gave oral informed consent to the study participation. Separate written consent was not required by the ethical committee. Moreover, the written information given to the participants stated that the participation in the study is voluntary, that by answering the questionnaires consent is given and consent can be withdrawn at any time.

### Study population and study design

**Fig 1:**
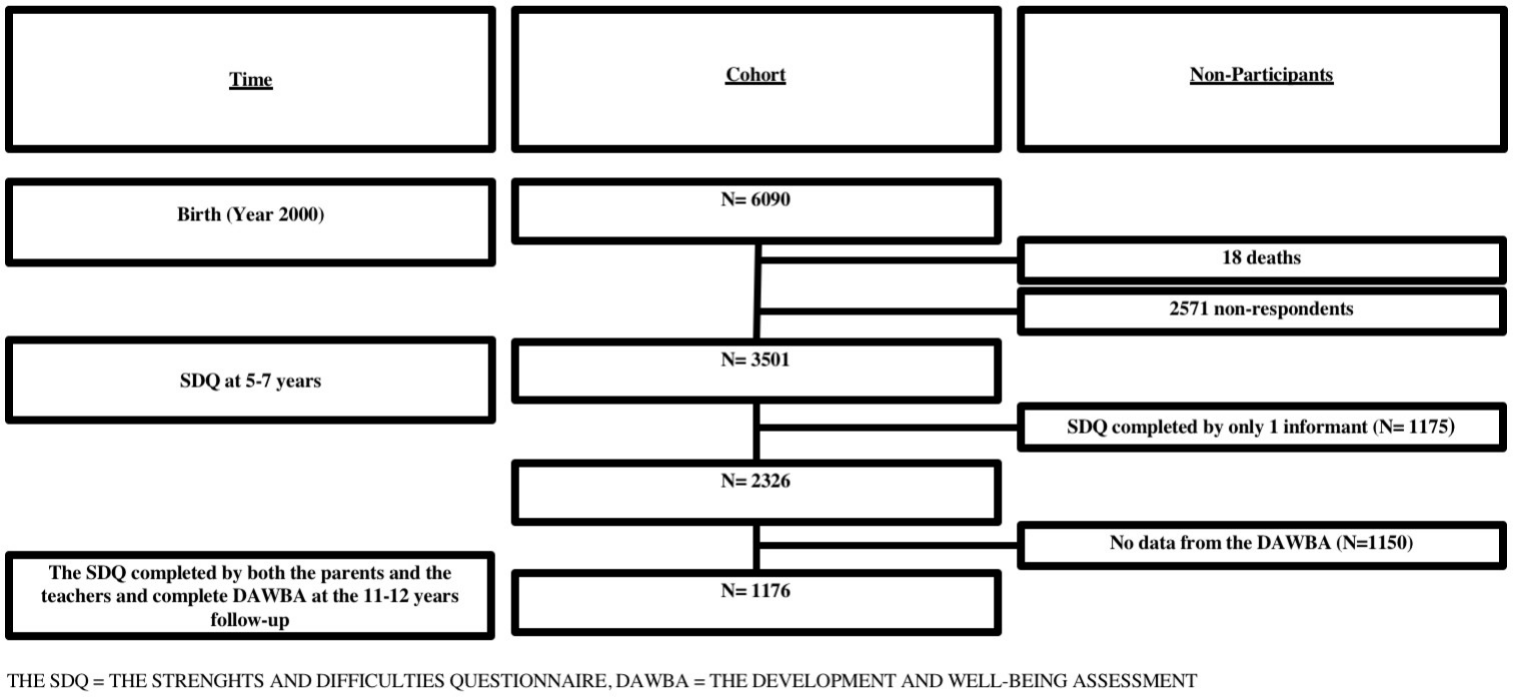
Flowchart for the study population.

The CCC2000 comprises all 6,090 children born in sixteen suburban municipalities surrounding the city of Copenhagen, Denmark, in the year 2000. Apart from an overrepresentation of ethnic minorities, the cohort is representative for children born in Denmark that year regarding key perinatal characteristics [27, 28]. In the years 2005–2007, the SDQ was completed for 3,501 children at age 5–7 years [29] with complete data from both teachers and parents for 2,326 children [26] (Fig 1). In year 2011–2012, 4,847 children of the cohort were eligible for follow-up, of whom a total of 2214 of children were diagnostically assessed using the Development and Well-Being Assessment (DAWBA), as detailed previously [30].

Children with full SDQ data from both teachers and parents at age 5–7 years and a diagnostic assessment of ICD-10 mental disorders at age 11–12 years comprised the population for this specific study corresponding to 1176 (1176/4847 = 24.3%) of the eligible sample.

### Recruitment process for the 5-7-year follow-up and the 11-12-year follow-up

For both the 5-7-year follow-up and the 11-12-year follow-up, the Danish civil register was used to obtain information about the children and their place of residence.

At the 5-7-year follow-up invitation letters were mailed to the parents who were asked to deliver the SDQ to the child’s preschool teacher. The paper-questionnaires were returned separately by the teachers and the parents [29].

At the 11-12-year follow-up, the families were invited to participate in the study by a letter and were contacted up to four times by mail or telephone. The parents and children were asked to answer web-based questionnaires using a personalized login, so that the parent and the child could answer the questionnaires independently. Furthermore, the children’s primary school teachers were emailed upon consent and contact information given by parents [30].

### Measures

#### The Strengths and Difficulties Questionnaire—The composition

The 25 SDQ questions inquire about both positive and negative characteristics [14] e.g. “*helpful if someone is hurt*, *upset or feeling ill”* and “*easily distracted*, *concentration wanders”*. The questionnaire can be completed by the parent(s), teacher and the children themselves above age 11 (self-rating not relevant for the current study). The questions are scored 0-1-2 on a Likert scale. For the scales regarding negative characteristics 0 = “not true”, 1 = “somewhat true” 2 = “certainly true”, while the scoring is reversed on the scales including positive characteristics (2 = “not true”, 1 = “somewhat true”, 0 = “certainly true”). The questions cover four domains of child and adolescent mental health difficulties corresponding to the four subscales: hyperactivity/inattention, conduct problems, emotional symptoms, and peer relationship problems. The pro-social problems are originally entered as positive filling questions, but the reverse subscale may capture autistics traits. Each subscale is rated as the sum score of five items, resulting in scores of 0 (min.) through 10 (max.) [8]. The impact supplement included in the extended version, consists of questions asking whether the respondent thinks the child/youth has a problem. If this is the case, the respondent is asked additional questions concerning overall distress and impairment [31]. The impact subscale measures the sum of distress and impact of problems at home, in school, among peers and in leisure time. The impact scores range from 0 to 6 for teachers and from 0 to 10 for parents.

#### The Strengths and Difficulties Questionnaire—The predictive algorithms

The SDQ predictive algorithms combine the symptom scale scores and the impact scores to predict ICD-10 diagnoses. Moreover, the SDQ predictive algorithms are predefined and consider both the internalizing and externalizing psychopathology and the impact of these problems in daily life and social functioning while integrating the perspectives of both the parents and the teacher. The SDQ diagnostic algorithm generates “unlikely”, “possible” or “probable” ratings for three categories of disorders: conduct disorders (SDQCD diagnostic algorithm), emotional disorders (SDQED diagnostic algorithm), hyperactivity-inattention disorders (SDQHK diagnostic algorithm), and for any psychiatric disorder (ANYDIAG diagnostic algorithm) combining the above three [11, 14, 29]. The pro-social behaviour scale and peer relationship scale do not contribute to the predictive diagnostic algorithms. The cut offs are based on the British population. For details on how the predictive algorithms are calculated and syntaxes for common statistical packages see http://www.sdqinfo.com/py/sdqinfo/c0.py.

In this study, we define screen-positive children by using the SDQ predictive algorithms with two different cut-points: lenient dichotomizing between unlikely and possible and strict dichotomization between possible and probable. In general, for a child being scored with a possible diagnosis requires a score above the 80^th^ percentile in a specific scale (e.g. hyperkinetic, conduct, or emotional) with concurrent impact score, whereas a probable diagnosis requires a score above the 90^th^ percentile. The specific rules for scoring the predictive algorithms including cut-offs and combining parent and teacher data, are presented in detail in the S1 Table–supplement algorithms.

#### The Development and Well-Being Assessment

The “Development and Well-Being Assessment” (DAWBA) is a comprehensive psychiatric interview for parents, teachers of children aged 5–16 years, which also includes information from children themselves from age 11. The DAWBA consists of structured questions covering the operationalized diagnostic criteria for International Classification Diseases– 10 (ICD-10) and Diagnostic and Statistical Manual for Mental disorders—IV (DSM-IV) diagnoses. Each of the sections are followed by open-ended questions for the respondent’s own description of problems [16]. The DAWBA-based ICD-10 diagnostic groups used in the current study are hyperkinetic-inattentive disorders, behavioral disorders and emotional disorders. Hyperkinetic-inattentive disorders include disturbance of activity and attention, hyperkinetic conduct disorder, other and unspecified hyperkinetic disorder and attention deficit disorder and behavioural disorders include oppositional defiant disorder and conduct disorder. Furthermore, emotional disorders include depressive episode, depressive disorder NOS, undifferentiated anxiety/ depression, separation anxiety disorder, specific phobia, social phobia, generalized anxiety and anxiety NOS including panic and agora. Moreover, a fourth variable of any mental disorder combining all three groups of ICD-10 diagnoses is used. The DAWBA has been shown to have good validity, for instance regarding the ability to discriminate between clinical and community samples [16]. Also, inter-rater reliability of the DAWBA-based diagnoses has been found good [32].

For the data collected for the 11-12-year follow-up, experienced child and adolescent psychiatrists, who were blinded for all other information, reviewed the results of the DAWBA diagnostic algorithms and the children’s, parents’ and teachers’ verbatim descriptions of the child’s problems to decide whether the child met the criteria for a specific mental disorder. Moreover, each case was rated independently by two consultant child and adolescent psychiatrist in pairs. Each pair consisted of one researcher and specialist of child and adolescence psychiatry and moreover one experienced child and adolescence clinician [30]. The three senior child and adolescent psychiatrists did the final classification with good inter-rater reliability (Kappa = 0.81) for the diagnostic groups [30].

### Statistical analysis

All the statistical analyses were performed using SPSS version 24. We used chi-square tests for categorical variables to compare the participants with all the non-participants still alive at the 11-12-year follow-up with regard to sociodemographic and perinatal characteristics.

The sensitivity, specificity, positive predictive value (PPV) and negative predictive value (NPV) were used to evaluate the predictive properties of the SDQ predictive algorithms defining possible and/or probable cases at age 5–7 years with regard to the prediction of the four categories of disorder: hyperkinetic-inattentive disorder, behavioural disorder and any mental disorder at age 11–12 years. We used logistic regression to determine ORs for each of the SDQ subscales with regard to the risk for having a diagnosis versus no disorder within each of the four diagnostic categories of disorders.

## Results

### Comparison of the participants and the non-participants

Among the 1176 participants, 611 (52.0%) were female and 565 (48.0%) were male. Attrition analysis shown in Table 1, showed that non-participants were more socio-economically disadvantaged for instance regarding household income.

**Table 1:** Characteristics of the participants compared to the non-participants.

Variable	Non-participants N = 4895 N (%)	Participants N = 1176 N (%)	*P* value	Missing
Sex				
Male	2550 (52.1)	565 (48.0)	0.013	0
Female	2345 (47.9)	611 (52.0)		
Birth weight(g)				
<2500	250 (5)	38 (3.3)	0.016	119
2500–4499	4396 (91.5)	1057 (92.2)		
≥4500	169 (3.5)	52 (4.5)		
Birth complications				
Yes	422 (8.6)	108 (9.2)	0.539	0
No	4473 (91.4)	1068 (90.8)		
Parity				
1	1720 (37.9)	419 (38.8)	<0.001	451
2	1903 (41.9)	484 (44.9)		
3	636 (14.0)	149 (13.8)		
4	282 (6.2)	27 (2.5)		
Maternal age at birth (years)				
16–20	215 (4.4)	10 (0.9)	<0.001	17
21–30	2574 (52.7)	550 (46.9)		
31–40	2028 (41.5)	593 (50.6)		
41–46	64 (1.3)	20 (1.7)		
Maternal education at birth (years)				
Low (0–10)	1231 (26.8)	120 (10.6)	<0.001	345
High (>10)	3363 (73.2)	1013 (89.4)		
Paternal age at birth (years)				
16–20	71 (1.5)	5 (0.4)	<0.001	120
21–30	1848 (38.6)	385 (33.1)		
31–40	2464 (51.5)	668 (57.4)		
41–50	364 (7.6)	96 (8.2)		
51–61	40 (0.8)	10 (0.9)		
Changes in family composition first year of life				
Yes	1506 (32.4)	261 (22.2)	<0.001	248
No	3143 (67.6)	913 (77.8)		
Household first income during first year of life				
1. quartile (low)	1360 (27.9)	149 (12.7)	<0.001	31
2nd quartile	1237 (25.4)	272 (23.2)		
3rd quartile	1180 (24.2)	332 (28.3)		
4th quartile (high)	1089 (22.4)	421 (35.9)		
Immigrant status				
2 parents born in Denmark	3331 (70.1)	946 (81.6)	<0.001	158
1 parent born in Denmark	571 (12.0)	121 (10.4)		
0 parents born in Denmark	852 (17.9)	92 (7.9)		
Parents living together at birth				
Yes	4442 (79.9)	1115 (88.3)	<0.001	17
No	439 (20.1)	58 (11.7)		

### The prevalence of ICD-10 mental disorders among the children in the 11-12-year follow-up

Among the 1176 participants, 89 (7.6%) were diagnosed with emotional disorders, 19 (1.6%) were diagnosed with behavioural disorders, and 31 (2.6%) were diagnosed with hyperkinetic-inattentive disorders. In total 123 (10.5%) children received at least one diagnosis within the three categories, and 15 of these, corresponding to 12.3%, had a diagnosis within two or three categories at the 11-12-year follow-up.

### The predictive value of the SDQ predictive algorithms in relation to the DAWBA-based ICD-10 diagnoses

A total of 222 (18.9%) children were screened as either a possible or probable case on at least one diagnostic algorithm (SDQHK, SDQCD and SDQED), and of these 54 (4.6%) children were screened positive on at least two subscales.

Table 2 displays the sensitivities, specificities, PPVs and NPVs for the SDQ predictive algorithms in relation to the DAWBA-based ICD-10 diagnoses 5–7 years later. Sensitivities ranging from 11.2–47.4, specificities ranging from 83.0–97.2, PPVs ranging from 5.0–28.9 and NPVs ranging from 91.6–99.0 were found for the possible and probable cases. For the probable cases sensitivities ranging from 4.5–21.1, specificities ranging from 96.8–99.5, PPVs ranging from 14.8–45.5 and NPVs ranging from 90.6–98.7 were found.

**Table 2:** Sensitivity, specificity, positive predictive value (PPV) and negative predictive value (NPV) for the four strengths and difficulties algorithms (5–7 years examination) in relation to the ICD-10 diagnoses (11–12 years follow-up).

	*Hyper-kinetic-inattentive disorders* N = 31/1176				*Behavioural disorders* N = 19/1176				*Emotional disorders* N = 89/1176				*Any mental disorder* N = 123/1176			
	Sens.	Spec.	PPV	NPV	Sens.	Spec.	PPV	NPV	Sens.	Spec.	PPV	NPV	Sens.	Spec.	PPV	NPV
**SDQHK**																
Possible/probable N = 45/ 1176	**41.9**	**97.2**	**28.9**	**98.4**	26.3	96.5	11.1	98.8	7.9	96.5	15.6	92.7	13.0	97.2	35.6	90.5
Probable N = 11/1176	**16.1**	**99.5**	**45.5**	**97.8**	5.3	99.1	9.1	98.5	2.2	99.2	18.2	92.5	4.9	99.5	54.5	90.0
**SDQCD**																
Possible/probable N = 179/1176	51.6	85.8	8.9	98.5	**47.4**	**85.3**	**5.0**	**99.0**	23.6	85.5	11.7	93.2	30.9	86.6	21.2	91.5
Probable N = 27/1176	32.3	98.5	37.0	98.2	**21.1**	**98.0**	**14.8**	**98.7**	4.5	97.9	14.8	92.6	9.8	98.6	44.4	90.3
**SDQED**																
Possible/probable N = 66/1176	25.8	94.9	12.1	97.9	31.6	94.8	9.1	98.8	**11.2**	**94.8**	**15.2**	**92.9**	13.8	95.3	25.8	90.5
Probable N = 20/1176	6.5	98.4	10.0	97.5	5.3	98.4	5.0	98.4	**4.5**	**98.5**	**20.0**	**92.6**	3.3	98.5	20.0	89.7
**ANYDIAG**																
Possible/probable N = 222/1176	58.1	82.2	8.1	98.6	52.6	81.7	4.5	99.1	29.2	82.0	11.7	93.4	**35.0**	**83.0**	**19.4**	**91.6**
Probable N = 51/1176	38.7	96.6	23.5	98.3	26.3	96.0	9.8	98.8	10.1	96.1	17.6	92.9	**13.8**	**96.8**	**33.3**	**90.6**

Sens. = sensitivity, Spec. = specificity, PPV = positive predictive value, NPV = negative predictive value, SDQHK = predictive algorithm for hyperactivity- inattentive disorders, SDQCD = predictive algorithm for conduct disorders, SDQED = predictive algorithm for emotional disorders, ANYDIAG = predictive algorithm for any psychiatric disorder

Table 3 shows the ORs for the SDQ predictive algorithms in relation to the DAWBA-based ICD-10 diagnoses. ORs ranging from 2.3–25.1 were found for the SDQ-based possible and probable cases with regard to the prediction of a corresponding diagnosis of a mental disorder 5–7 years later (e.g. SDQHK predicting hyperkinetic disorder). For the probable cases ORs in the range 3.2–36.5 were found. Overall high ORs were found for the SDQHK diagnostic algorithm in relation to the diagnosis of hyperkinetic-inattentive disorder. The ORs found for the SDQED algorithm in relation to emotional disorders were generally lower than the ORs found for the SDQHK in relation to hyperkinetic-inattentive disorder. The ORs found for the SDQCD diagnostic algorithm in relation to the diagnosis of behavioural disorders were lower as well. For the diagnostic algorithm combining all three disorders, ANYDIAG, the highest OR found was 4.8 (95% CI 2.6–8.9) for probable cases based on the SDQ predictive algorithms with regard to the corresponding DAWBA-based diagnosis “any mental disorder” (including hyperkinetic-inattentive disorder, behavioural disorder and emotional disorder).

**Table 3:** The odds ratios (ORs) for the four Strengths and Difficulties Questionnaire algorithms (5–7 years examination) in relation to the ICD-10 diagnoses (11–12 years follow-up).

	*Hyperkinetic-inattentive disorders*		*Behavioural disorders*		*Emotional disorders*		*Any mental disorder*	
	N = 31/1176		N = 19/1176		N = 89/1176		N = 123/1176	
	OR	95% CI	OR	95% CI	OR	95% CI	OR	95% CI
**SDQHK**								
Possible/probable N = 45/1176	**25.1**	**11.3–55.6**	10.0	3.4–29.0	2.4	1.0–5.4	5.3	2.8–10.0
Probable N = 11/1176	**36.5**	**10.5–127.3**	6.4	0.8–52.4	2.8	0.6–12.9	10.8	3.23–35.8
**SDQCD**								
Possible/probable N = 179/1176	6.4	3.1–13.3	**5.2**	**2.1–13.1**	1.8	1.1–3.1	2.9	1.9–4.4
Probable N = 27/1176	31.6	12.9–77.1	**13.2**	**4.1–42.7**	2.2	0.7–6.4	7.5	3.4–16.4
**SDQED**								
Possible/probable N = 66/1176	6.5	2.8–15.2	8.4	3.1–23.0	**2.3**	**1.2–4.7**	3.3	1.8–5.9
Probable N = 20/1176	4.3	1.0–19.5	3.3	0.4–26.2	**3.2**	**1.0–9.6**	2.2	0.7–6.6
**ANYDIAG**								
Possible/probable N = 222/1176	6.4	3.1–13.2	5.0	2.0–12.3	1.9	1.2–3.0	**2.6**	**1.7–3.9**
Probable N = 51/1176	17.9	8.1–39.5	8.6	3.0–25.0	2.8	1.3–6.0	**4.8**	**2.6–8.9**

OR = Odds Ratio, CI = Confidence Interval, SDQHK = predictive algorithm for hyperactivity-inattention disorders, SDQCD = predictive algorithm for conduct disorders, SDQED = predictive algorithm for emotional disorders, ANYDIAG = predictive algorithm for any psychiatric disorder

High ORs were also found across the diagnostic categories. For instance, high ORs were found for the SDQHK diagnostic algorithm for possible and probable cases in relation to a later diagnosis of behavioural disorders (OR 10.0, 95% CI 3.4–29.0), and vice versa, the SDQCD diagnostic algorithm for probable cases in relation to a later diagnosis of hyperkinetic disorders was very high (OR 31.6, 95% CI 12.9–77.1). For the SDQED diagnostic algorithm for possible and probable cases high ORs were found in relation to a later diagnosis of behavioural disorders (8.4, 95% CI 3.1–23.0) and of hyperkinetic-inattentive disorders (6.5, 95% CI 2.8–15.2) compared to emotional disorders (2.3 95% CI 1.2–4.7).

## Discussion

### Main results

To our knowledge, this study is the first to examine the predictive validity of the SDQ predictive algorithms in a longitudinal study across a broad range of mental disorders. Children who were rated as either possible or probable on the predictive algorithms in preschool age, had a markedly greater risk of having a mental disorder in preadolescence. Particularly high ORs were found when using the predictive algorithms for the prediction of hyperkinetic-inattentive disorders and for the prediction of behavioural disorders, while the ORs found for the prediction of emotional disorders were lower. In general, using “probable” ratings on all predictive algorithms yielded better ORs, illustrating that children with the highest scores were indeed at higher risk.

The sensitivities found were moderate for hyperkinetic/inattention disorders, but relatively low for emotional disorders, while the PPVs were low as well, particularly for behavioural disorders. The specificities and NPVs found were moderate to high.

### Strengths and limitations

Important strengths of the study include the long follow-up period of 5–7 years and the large study population. A rigorous diagnostic assessment was conducted using the DAWBA at the 11-12-year follow-up, synthesizing both a highly structured interview and open-ended questions reviewed and diagnosed by experienced clinicians with high reliability [30]. We also included information from both parents and teachers, which is essential in screening for mental health problems in childhood [9–11].

Interpretation should consider potential limitations of the study. First, the exact cutoff values used in the SDQ predictive algorithms are based on the British population. However, Danish norms for the SDQ scales found that the 80- and 90- percentile cutoffs for the SDQ scales are in the same range as the British cutoffs [33]. Second, children not participating in the study were more often from families of lower socio-economic disadvantage, more often had immigrant background and more often had low birthweight compared to the participants. Socioeconomic disadvantage is well known to be associated with mental health problems[34], low birth weight is associated with developing mental health problems [35], while a recent review has shown that first generation migrants have an increased risk of developing mental disorders [36].Thus, children at higher risk of mental disorder in preadolescence might be underrepresented among the participants. As a consequence, we assume less variation with regard to both the exposure and outcome variables, and consequently the estimated odds ratios reported are likely attenuated. Third, we cannot reject the possibility of informant bias as parents were informants in both the 5-7-year follow-up and in the 11-12-year follow-up. This bias might, however, be reduced because of the long-time span between the two follow-ups. Moreover, the children themselves, and in some cases the school teachers, also contributed to the DAWBA diagnoses at 11–12 years. Fourth, we cannot detect which children would have fulfilled criteria for a diagnosis at some point in between the follow-ups. Some children may have received successful treatment for a disorder between the follow-ups and hence no longer fulfilled criteria at the DAWBA assessment. Hence, the predictive values may have been attenuated because of the 6-year time lag between the two points of assessments.

### Interpretation

The SDQ predictive algorithms identified children with a significantly greater risk of being diagnosed with a mental disorder in preadolescence. The findings are in accordance with the patterns described in previous research. It has been described that the emotional disorders most often have onset during childhood or adolescence [2], but with a sharp rise in incidence in adolescence [37] and that the course of emotional disorders has been found to be less stable than for other neurobehavioral disorders [2]. Additionally, previous research has suggested that onset of conduct disorders often occurs in childhood as well as in adolescence, whereas ADHD has an early neuro-developmental origin [38]. Hence, the high predictive values found for hyperkinetic-inattentive disorders, the intermediate predictive values for behavioural disorders and the lower values found for the prediction of emotional disorders were expected. Also, strong associations between SDQ-predictions at 5–7 years and diagnoses at 11–12 years were not only found within the same diagnostic category (homotypic course) but also across the full range of ICD-10 diagnoses (heterotypic course). For example, a large overlap between hyperkinetic- and behavioural disorders were found, corresponding well with previous research [2, 39].

Using the SDQ predictive algorithms by combining the symptoms and their everyday impact, is one way to take advantage of the SDQ and utilize multiple informants comprehensively and furthermore one way to imitate the clinical process. The SDQ predictive algorithms have been well established in cross-sectional studies [11, 14, 15, 18, 19, 40], and our findings suggest that they could advantageously be used as part of a screening program to identify children at markedly increased risk of mental health diagnoses later in pre-adolescence. Such screening could potentially decrease the treatment delay and the gap between the numbers of children in the general population meeting criteria for mental disorders and the numbers of children who actually receive treatment in community or mental health services [4]. Because of the strong association found between having persistent or multiple episodes of mental disorder in adolescence and developing a mental disorder in young adulthood [3], instruments are needed to identify the children and adolescents with mental health problems. The SDQ is a brief and easy-to-read screening instrument and utilizing the SDQ predictive algorithms could be a cost-efficient way to identify children that can benefit from more extensive screening and perhaps benefit from indicated preventive measures [41].

The sensitivities and PPVs found in the current study were lower than the predictive values found in the original cross-sectional study for the SDQ predictive algorithms [11]. The developmental factors and instability of psychopathology from childhood to preadolescence naturally affected the sensitivities and PPVs found. This underlines that especially emotional problems should be continuously assessed throughout childhood and that screening with the SDQ predictive algorithms cannot stand alone. Furthermore, effective preventive interventions need to be in place if population- and/or indicated screening is to be meaningfully established.

## Supporting information

S1 Table Supplement algorithms. (DOCX)
