# Supplementary material for: The predictive validity of the Strengths and Difficulties Questionnaire in preschool age to identify mental disorders in preadolescence
Source: PLoS One. 2019 Jun 3;14(6):e0217707. doi: 10.1371/journal.pone.0217707 (PMC6546211; doi:10.1371/journal.pone.0217707)
Supplement: S1 Table — (DOCX) [file pone.0217707.s001.docx]

**Supplement algorithms**

**S1 Table 1. Supplement Algorithms – Description of the cut-offs behind the predictions of unlikely/possible/probable diagnoses according to the diagnostic algorithms of the Strengths and Difficulties Questionnaire, combining parent and teacher ratings**

| **Cut offs for possible or probable ratings of diagnosis for parents/teachers** | | | | |
| --- | --- | --- | --- | --- |
|  | **Parent** | **Teacher** |  |  |
| Hyperkinetic disorder unlikely | Hyperactivity subscale <6 and/or impact<1 | Hyperactivity subscale ≤6 and/or impact<1 |  |  |
| Hyperkinetic disorder possible | Hyperactivity subscale ≥6 and Impact≥1 | Hyperactivity subscale ≥6 and Impact ≥1 |  |  |
| Hyperkinetic disorder probable | Hyperactivity subscale ≥7 and Impact>2  *OR*  Hyperactivity subscale ≥9 and Impact ≥1 | Hyperactivity subscale ≥7 and Impact≥2 |  |  |
| Conduct disorder unlikely | Conduct subscale <4 | Conduct subscale <3 |  |  |
| Conduct disorder possible | Conduct subscale ≥4 | Conduct subscale >3 |  |  |
| Conduct disorder probable | Conduct subscale ≥5 and impact ≥2 | Conduct subscale ≥4 and impact ≥2 |  |  |
| Emotional disorder unlikely | Emotional subscale <5 and/or impact <1 | Emotional subscale <5 and/or impact <1 |  |  |
| Emotional disorder possible | Emotional subscale ≥5 and impact ≥1 | Emotional subscale 5 and impact ≥1 |  |  |
| Emotional disorder probable | Emotional subscale ≥6 and impact ≥2 | Emotional subscale 6 and impact≥2 |  |  |
| **Combining parent and teacher ratings for possible or probable diagnosis** | | | |  |
| Hyperkinetic disorder unlikely | If parent AND teacher = unlikely  *OR*  If parent OR teacher = possible BUT the other rater = unlikely | |  |  |
| Hyperkinetic disorder possible | If parent AND teacher = possible  *OR*  If parent OR teacher = probable BUT the other rater =unlikely | |  |  |
| Hyperkinetic disorder probable | If parent OR teacher = probable AND the other rater = possible/probable | |  |  |
| Conduct disorder unlikely | If parent AND teacher = unlikely | |  |  |
| Conduct disorder possible | If parent OR teacher = possible | |  |  |
| Conduct disorder probable | If parent OR teacher = probable | |  |  |
| Emotional disorder unlikely | If parent AND teacher = unlikely | |  |  |
| Emotional disorder possible | If parent OR teacher = possible  *OR*  If parent OR teacher = probable AND if Hyperkinetic/Conduct disorder = probable | |  |  |
| Emotional disorder probable | If parent OR teacher = probable AND Hyperkinetic/Conduct disorder ≠ probable | |  |  |
